# Investigation of Buckling Behaviors in Carbon Nanorings Using the Chebyshev–Ritz Method

**DOI:** 10.3390/nano14231868

**Published:** 2024-11-21

**Authors:** Xiaobo Wang, Guowen Kuang, Hongmei Tian, Zhibin Shao, Ning Dong, Tao Lin, Li Huang

**Affiliations:** 1 Physics Laboratory, Industrial Training Center, Shenzhen Polytechnic University, Shenzhen 518055, China; hmtian@szpu.edu.cn (H.T.); zhibin_shao@szpu.edu.cn (Z.S.); dongning@szpu.edu.cn (N.D.); 2 Institute of Applied Artificial Intelligence of the Guangdong-Hong Kong-Macao Greater Bay Area, Shenzhen Polytechnic University, Shenzhen 518055, China; 3 College of New Materials and New Energies, Shenzhen Technology University, Shenzhen 518118, China; lintao@sztu.edu.cn; 4 Department of Physics, Southern University of Science and Technology, Shenzhen 518055, China; huangl@sustech.edu.cn

**Keywords:** carbon nanoring, molecular dynamics simulation, continuum mechanical modeling, Chebyshev–Ritz method

## Abstract

Carbon nanorings (CNRs) serve as an ideal quantum system for novel electronic and magnetic properties. Although extensive theoretical studies utilizing molecular dynamics (MD) simulations have investigated the formation and structural characteristics of CNRs, systematically analyzing their properties across various toric sizes remains challenging due to the inherent complexity of this system. In this study, we introduce a novel finite element method, the Chebyshev–Ritz method, as an alternative approach to investigating the structural properties of CNRs. Previous MD simulations demonstrated that stable CNRs adopt a regular buckled shape at specific toric sizes. By meticulously selecting mechanical parameters, we observe that the critical deformation of a CNR with 50 repeated units, as determined by the Chebyshev–Ritz method, aligns with an MD simulation presenting a buckling number of 14. Additionally, the implementation of the Chebyshev–Ritz method with a constant mechanical parameter for 50 repeated units reveals a structural transition at varying toric sizes, leading to the stabilization of buckling numbers 13, 14, and 15. This structural transition across different buckling modes has also been corroborated by MD simulations. Our approach offers a reliable and accurate means of examining the structural properties of large-scale nanomaterials and paves the way for further exploration in nanoscale mechanics.

## 1. Introduction

Carbon nanorings (CNRs), as a variant of carbon nanotubes (CNTs), were first observed by Liu et al. while studying laser-grown single-walled CNT materials [1]. These circular structures can be synthesized through various experimental methods and usually contain multiple fibers, which may be formed from single-walled, double-walled, or multi-walled CNTs [2]. The diameters of CNRs range from several hundred nanometers, with widths typically around ten nanometers, varying depending on experimental synthesis methods [2,3]. Due to their ring-like morphology, CNRs are expected to exhibit distinctive features compared to their rod-like counterparts [2,3,4]. In terms of magnetic properties, quantum transport measurements have demonstrated that CNRs exhibit negative magnetoresistance to weak one-dimensional localization at low temperatures [5], while thicker CNRs show a stronger magnetic response, as observed in phase shift analysis using magnetic force microscopy [6]. The Aharonov–Bohm effect [7] magnetically induces current densities in CNRs with different shapes [8], and the magnetization behavior of CNRs with surface anisotropy [9] has also been theoretically investigated by density functional theory (DFT) and tight-binding modeling methods. These findings suggest that CNRs are promising as an ideal quantum system for exploring novel electronic and magnetic properties.

Despite these advances, most studies concerning the electronic and magnetic properties of CNRs have relied on idealized models of defect-free or polygonal, circular quantum wires [7,8,9,10,11]. However, the experimentally synthesized CNRs usually consist of multiple fibers. Although single-walled CNRs have been successfully synthesized, controlling their size remains challenging, and the critical diameters of single-walled CNRs are limited to below one hundred nanometers [12,13]. Producing single-walled CNRs with smaller sizes experimentally continues to be difficult. Nonetheless, extensive theoretical analysis has been conducted on the formation and structural characteristics of CNRs. Molecular dynamics (MD) simulations have revealed that CNRs often adopt a regular buckled shape on their inner surface [14,15]. Similar morphologies have been observed in zinc oxide nanorings, which form through the self-coiling of nanobelts [16]. Some researchers have focused on the fact that the stable ring adopts a regular polygonal shape [17]. Additionally, MD methods have also been used to examine the thermal stability and morphological variations in CNRs with different diameters during annealing processes [18]. The energy and structural stability of CNRs and CNR-based nanochain structures have also been addressed in MD studies [19,20,21,22,23,24]. The buckling behaviors of CNRs have been investigated by employing the condensed-phase optimized molecular potential for atomistic simulation studies (COMPASS), revealing the corresponding critical tension displacements and buckling shapes [25]. Furthermore, the mechanical properties of CNRs have been explored using the ReaxFF force field to estimate their Young’s modulus, extensibility, and tensile strength [26].

Although MD simulations can provide valuable structural insights, they face significant limitations when systematically investigating the structural properties of CNRs with different toric sizes. Adjusting simulation parameters with a continued change to explore a wide range of configurations leads to substantial computational costs and complexity. The more severe problem is that these systems are prone to becoming trapped in the local minimum of metastable high-energy states due to the strong directional bonding of carbon atoms, resulting in large energy barriers between various metastable structures. To address these challenges, continuum mechanical modeling (CMM) emerges as a desirable alternative. CMM simulations offer high computational efficiency, provide a holistic view of nanostructures, and allow for the straightforward analysis of complex systems, which is difficult to achieve with MD simulation. In this study, we introduce a novel approach to CMM, the Chebyshev–Ritz method, to analyze the structural properties of CNRs. The Ritz method, a finite element displacement method, is widely used in the vibration analysis of structural elements due to its simplicity and high accuracy [27,28,29]. However, the accuracy and applicability of the Ritz method are significantly dependent on the choice of admissible functions used in the analysis [30]. Building on this, D. Zhou et al. developed a method using Chebyshev polynomials and trigonometric functions to construct admissible functions that satisfy the geometric boundary conditions of the corresponding structures [30,31,32,33]. This approach was demonstrated to provide a precise description of the vibration of structural elements with both rapid convergence and high accuracy. While the advantages of CMM in investigating the structural properties of nanomaterials are evident, its validity requires further verification through direct comparison with molecular dynamics or ab initio simulations [34]. Therefore, this work aims to verify the effectiveness of the Chebyshev–Ritz method in investigating the structural properties of CNRs. We conducted a systematic comparison using MD simulations, focusing on an extensively investigated structural problem where CNRs exhibit highly regular buckled shapes on their inner surface under specific toroidal parameters. We found that both the MD simulations and the Chebyshev–Ritz method capture the regular buckled structures of CNRs. Unlike MD simulations, which are often limited by computational constraints on system size, the Chebyshev–Ritz method can efficiently handle systems of arbitrary dimensions. This flexibility enables it to deduce mechanical properties that align well with experimental observations, offering a robust alternative for studying large-scale nanomaterials.

## 2. Theoretical Methods

**Figure 1:**
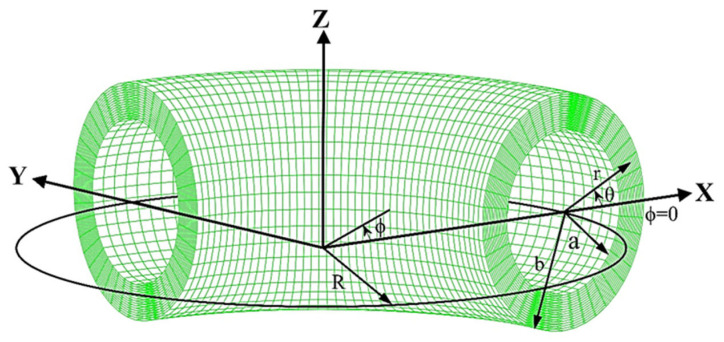
A hollow ring structure with an azimuthal cross-section and the corresponding coordinate systems in the *r*, *θ*, and *ϕ* directions.

CNRs are modeled as a ring with an azimuthal cross-section, as illustrated in Figure 1. The radii of the inner and outer cross-sections are denoted as *a* and *b*, respectively, to model single- or multi-walled CNRs. The corresponding centroidal-axis radius is *R*. To describe the strain and stress of the ring, we employ a coordinate system that combines the polar coordinate (*r*, *θ*) of each cross-section and the circumferential coordinate *ϕ* around the ring.

In this fixed coordinate system, the relationships between three-dimensional tensorial strain components and displacement components in the orthogonal curved coordinate system are defined by

(1) $$\varepsilon_{\mathrm{rr}}=\frac{\partial u_{r}}{\partial r},\;\;\;\varepsilon_{\theta \theta }=\frac{1}{r}\frac{\partial u_{\theta }}{\partial \theta }+\frac{u_{r}}{r}\;\varepsilon_{\phi \phi }=\frac{1}{R+r\cos\theta }\frac{\partial u_{\phi }}{\partial \phi }+\frac{\cos\;\theta }{R+r\cos\;\theta }u_{r}-\frac{\sin\;\theta }{R+r\cos\theta }u_{\theta }\;\gamma_{r\theta }=\frac{\partial u_{\theta }}{\partial r}-\frac{u_{\theta }}{r}+\frac{1}{r}\frac{\partial u_{r}}{\partial \theta }\;\gamma_{\theta \phi }=\frac{1}{r}\frac{\partial u_{\phi }}{\partial \theta }+\frac{\sin\;\theta }{R+r\cos\;\theta }u_{\phi }-\frac{1}{R+r\cos\;\theta }\frac{\partial u_{\theta }}{\partial \phi }\;\gamma_{\phi r}=\frac{1}{R+r\cos\;\theta }\frac{\partial u_{r}}{\partial \phi }+\frac{\partial u_{\phi }}{\partial r}-\frac{\cos\;\theta }{R+r\cos\;\theta }u_{\phi }$$

where $u_{r}$, $u_{\theta }$, and $u_{\phi }$ are the displacements in the *r*, *θ*, and *ϕ* directions, respectively. To establish the correlation between the Chebyshev–Ritz method and the MD simulation results (discussed later), we define a buckling number, *n* (where *n* ≥ 2), to describe the number of buckles in the regular buckled shapes of the CNRs observed in the MD simulations. These structures exhibit *C_nh_* symmetry, dominated by the geometrical periodicity in the circumferential *ϕ* directions. The buckling number, *n*, is incorporated into the displacement function, which is expanded using Chebyshev polynomials and trigonometric functions. This number, originally defined as the circumferential wave number, must be an integer to ensure the periodicity of the circumferential symmetry [30]. Increasing the buckling number corresponds to increasing the strain load in the circumferential *ϕ* direction. To facilitate calculations, we rescale the coordinates using *h* = (*b* − *a*)/2 and *δ* = (*b* + *a*)/2, leading to the rescaled coordinate $\overset{-}{r}\;$ = (r− *δ*)/*h*. The determinant of the Jacobian matrix of the coordinate system is |*J*| = r(*R* + r*cos θ*). Since the elastic properties of a two-dimensional hexagonal structure are isotropic [27], the strain energy, V, of an isotropic elastic body with a ring structure can be expressed by the following integral over the bulk domain:

(2) $$\begin{array}{c} \;\;\;\;\;\;\;V=\mathrm{hG}\;\int_{0}^{2\pi }\int_{-1}^{1}\left[(1+\lambda )(\varepsilon_{rr}^{2}+\varepsilon_{\theta \theta }^{2}+\varepsilon_{\phi \phi }^{2}\right)\\ \;\;\;\;\;\;\;\;+2\lambda (\varepsilon_{rr}\varepsilon_{\theta \theta }+\varepsilon_{\theta \theta }\varepsilon_{\phi \phi }+\varepsilon_{\phi \phi }\varepsilon_{rr})\;\;\;\\ \;\;\;\;\;\;\;\;\;\;+\frac{1}{2}(\varepsilon_{r\theta }^{2}+\varepsilon_{\theta \phi }^{2}+\varepsilon_{\phi r}^{2})]\bar{r}(R\;+\bar{r}\cos\theta )d\bar{r}d\;\theta \end{array}$$

where *λ* and *G* represent the parameters of the Lamé constants for an isotropic material, which can be expressed in terms of the Young’s modulus and Poisson’s ratio. In our static analysis, we focus on finding the equilibrium configurations of the CNRs by applying the displacement-based extremum energy principle to the strain energy, *V* [30]. Since we set the angular frequency to be zero, the kinetic energy becomes zero and does not contribute to the Lagrangian.

The MD simulation is carried out using the Large-scale Atomic/Molecular Massively Parallel Simulator (LAMMPS) package, employing the COMPASS to describe the interactions of carbon atoms in the CNRs. The COMPASS is an ab initio force field designed for simulating and predicting the gas-phase properties (structural, conformational, vibrational, etc.) of molecules and polymers [35]. It has been extensively used to study the mechanical properties of CNTs, effectively capturing behaviors such as buckling and variations in the Young’s modulus, as demonstrated in previous studies [36,37,38,39]. Therefore, it is reasonable to use this force field to study CNRs in molecular dynamics calculations. Details regarding the force field method and model parameters associated with carbon–carbon bonds are available in previous reports [35]. In the MD simulations, we use the velocity Verlet integration method with a time step of 1 fs. The ensemble maintains a constant temperature and volume by controlling the temperature (NVT ensemble). The initial particle velocities are randomly assigned based on the Maxwell–Boltzmann distribution. The Nosé method is employed for temperature control. Simulations are carried out at 1 K to minimize thermal effects, ensuring compatibility with the CMM Chebyshev–Ritz method.

## 3. Results and Discussion

### 3.1. MD Simulations of CNRs Exhibiting Regular Buckled Shapes and Those Without

To create defect-free CNRs, we fold the ends of a straight, defect-free CNT to form a circular ring through covalent bonding [12,13]. This method avoids boundary ambiguities and additional defects such as pentagons or heptagons. In our research, we use the (7, 7) armchair CNTs, bending them into closed rings to serve as the initial configurations for our MD simulations. The centroidal-axis radius, *R*, of the ring corresponds to the length of the CNTs, while the cross-section diameter is maintained at 9.49 Å. The repeat unit of the CNTs is 2.46 Å, and the length of the CNRs is defined as *l* = 2*πR*, which is an integer multiple of the repeat unit. It is important to note that this folded configuration is not a minimum-energy structure. This is due to the tension caused by the contraction of C–C bonds on the inner radius, combined with the lack of compensatory expansion on the outer radius where the C–C bonds are stretched.

Initially, MD simulations were conducted to examine the structural properties of CNRs with two distinct lengths, characterized by 50 and 200 repeated units. The Chebyshev–Ritz method can be considered a low-energy phonon approximation of MD simulations at zero temperature. This approach simplifies complex atomic interactions by treating the CNRs as a continuous medium, effectively addressing vibration issues. It is particularly effective for studying low-frequency, long-wavelength vibration modes, which correspond to low-energy states in the phonon spectrum [40,41]. To compare these results with our MD simulations, the temperature was set to 1 K using the NVT ensemble to minimize thermal effects. Subsequently, we maintained a consistent toric size but adjusted the Poisson’s ratio and Young’s modulus of the CNRs within the Chebyshev–Ritz framework. These adjustments are necessary to align the results of the Chebyshev–Ritz method with those of the MD simulations, specifically ensuring both methods predict the same buckling number for CNRs with 50 repeated units.

**Figure 2:**
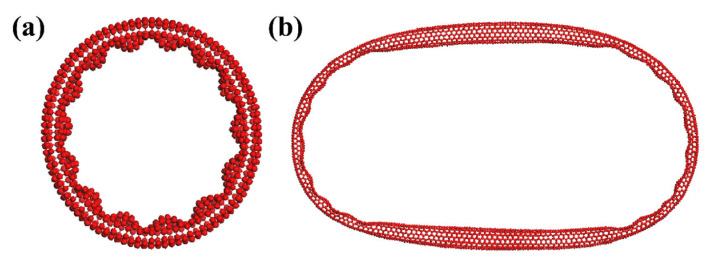
Equilibrated morphologies of CNRs with (**a**) 50 and (**b**) 200 repeated units showing the buckling on the inner sides of the toroids.

The equilibrated structure of a CNR with 50 repeated units is depicted in Figure 2a. Throughout the dynamic process, the perfect ring structure does not represent a minimum on the potential energy surface due to the uneven tension distribution across the inner and outer sides of the ring. Consequently, the bonds experience significant vibrations, eventually leading to the spontaneous formation of buckles that lower the energy. The final equilibrated structure, as shown in Figure 2a, clearly shows a regular buckled shape. CNRs exhibit a regular buckled configuration that represents a critical symmetry-breaking event. This transition disrupts the continuous rotational symmetry of the ring, shifting it from a high-symmetry structure, *C_∞h_*, to a lower-symmetry one, *C_nh_*. In earlier research, Yakobson et al. explored how CNTs subjected to significant deformations could reversibly transition into various morphological patterns, which behaved as abrupt singularities on the strain energy curve corresponding to the loss of different symmetry forms [42]. We define the buckling number to describe regular buckled configurations with *C_nh_* symmetry, as indicated in Figure 2a. The buckling number for the CNR with 50 repeated units is determined to be 14, where 14 lobes are visible in the inner circumference, with the tension predominantly concentrated within these buckles. Geometrical measurements reveal that the sizes of the 14 buckles are nearly identical, with the measured atomic difference being less than 0.8 Å in the direction of the centroidal axis of the ring, demonstrating the uniformity of the buckling throughout the structure.

Our MD simulations, along with previous research, have uncovered a family of nanostructures with highly regular buckled shapes, resulting from the varying tensions across the inner and outer regions of CNRs at a specific radius [14,15]. However, previous experimental studies have not detected such configurations in isolated CNRs [1,2,43]. Experimentally synthesized CNRs typically have a large radius, around hundreds of nanometers, displaying a smooth toroidal form with multiple layers of nanofibers held together by van der Waals forces. Within such a large radius, detecting regular buckled structures experimentally is challenging, as the local bending curvature resembles that of a cylindrical tube. As shown in Figure 2b, achieving a regular buckled shape with a consistent buckling number is difficult when CNRs have a length equivalent to 200 units. The equilibrium configuration reveals an irregular shape with randomly distributed kinks. At this scale, maintaining equilibrium between a constant buckling number and the expansive length of the CNRs is challenging, similar to previous observations [15,18]. Experimentally, detecting such regular buckled configurations in CNRs requires reducing the radii and synthesizing CNRs with only a few layers, or potentially a single layer. Unfortunately, CNRs with diameters of only a few tens of nanometers have not yet been observed in these experiments.

### 3.2. Regular Buckled Shape Achieved by Chebyshev–Ritz Method

CNRs are also considered a deformed variant that arises from the synthesis process of CNTs, initially identified in experiments with laser-grown single-walled CNTs. A potential intrinsic link is the notion that CNRs may form by folding CNTs during their growth process. Sano et al. demonstrated that CNRs can be directly produced from CNTs with chemically functionalized ends—a process similar to folding the tubes into ring structures in the solution, as described in [13]. Consequently, understanding the mechanical deformation of CNTs under compression could provide valuable insights into the structural properties of CNRs with small radii. Furthermore, experimental studies have revealed that when single- or multi-walled CNTs are embedded within a polymeric film and subsequently stressed, they tend to collapse, creating local rippled buckled structures on the inner side of the bending plane to release tension [44]. Poncharal et al. observed that the elastic bending modulus of CNTs, which depends on the tube’s diameter, decreases markedly as the diameter increases [45]. This indicates a transition from a uniform elastic response to one characterized by wave-like distortions when the CNTs are bent. Such structural responses closely resemble the regular buckled configuration observed in our MD simulations. Compared to uniform bending, the appearance of these regular buckles is associated with the reduced compression in the C-C bonds located at the inner arc of the bend, resulting in a considerable reduction in strain energy.

**Figure 3:**
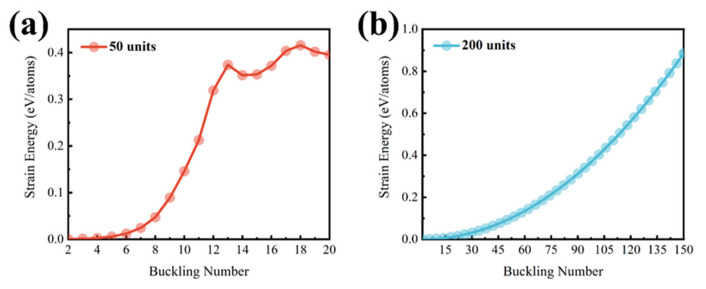
The strain energy per atom calculated by the Chebyshev–Ritz method at lengths of (**a**) 50 units and (**b**) 200 units.

By adjusting the Poisson’s ratio and Young’s modulus, our Chebyshev–Ritz model yields results comparable to MD simulations. In our MD simulations, the ring structure adopted a regular buckled shape at a length of 50 units. To compare the results from these two methods, the mechanical parameters of the system are normalized. It is found that the Chebyshev–Ritz method agrees well with the MD simulation when the parameters are set to a Poisson’s ratio of *v* = 0.178 and a Young’s modulus of *Y* = 17.1 TPa. These values are consistent with the mechanical properties reported for single-walled carbon nanotubes in the literature [46,47,48,49]. While exact values can vary depending on specific CNT structures and measurement methods, our chosen parameters fall within the commonly accepted ranges, ensuring the physical reasonableness of our model. The internal and external radii are set to *a* = 5 Å and *b* = 5.667 Å, respectively. Figure 3a illustrates the relationship between the strain energy per atom and the buckling number for a CNR with 50 repeated units, as calculated using the Chebyshev–Ritz method. The curve shows that at small buckling numbers, the strain energy per atom increases with the buckling number, indicating increased deformation within the nanoring. Notably, there is a local minimum in the strain energy per atom at a buckling number of 14, suggesting a significant change in the structural response of the nanoring. This local minimum represents a sudden change in the curve, indicating a structural transition into a stable buckling mode, which aligns well with our MD simulations. Beyond this local minimum, the strain energy per atom varies differently with the buckling number, reflecting the nanoring’s response to further deformation modes.

It is important to note that in our CMM simulations using the Chebyshev–Ritz method, the strain energy plotted in Figure 3a represents the additional strain energy due solely to buckling deformations beyond the inherent bending strain energy of the nanoring. The inherent strain energy resulting from bending a straight carbon nanotube into a ring is present even in the unbuckled state (n=0) and is considered part of the reference configuration; thus, it is not included in strain energy. Our primary interest lies in understanding the mechanical instabilities and identifying the critical points where sudden changes in the buckling mode occur, which are represented by local extrema in the energy landscape corresponding to structural phase transitions and stable configurations under specific constraints. This focus is similar to the analysis of abrupt transitions in deformation modes observed in the stress–strain curves of carbon nanotubes, as discussed in Yakobson’s work [43]. Therefore, the mechanical instabilities and transitions between different buckling modes of nanorings are reflected by the sudden changes in the strain energy versus buckling number curve, indicated by the local minima, rather than solely seeking the lowest energy configurations as in MD simulations. The apparent discrepancy between our method and MD simulations arises from the different assumptions and objectives of the two modeling approaches. In the Chebyshev–Ritz method, the displacement components are expanded using Chebyshev polynomials and trigonometric functions involving the buckling number, n, as described in the Methods section. The strain components are functions of the spatial derivatives of the displacement components. When n is small, the spatial variation in the displacement field due to buckling is minimal, leading to smaller strain components and, consequently, a small increase in strain energy. As n increases, the deformation becomes more significant, resulting in larger strain components and a higher strain energy.

Building on this understanding, we observe that for CNRs with larger lengths, such as those with 200 repeated units, the strain energy per atom calculated by the Chebyshev–Ritz method displays a smooth curve without any local minima, even when the buckling number reaches 150, as shown in Figure 3b. For CNRs of this length, it is reasonable to truncate the buckling number at 150, as searching for local minima at higher values is unnecessary. This decision is based on the actual size of typical buckles. MD simulations for a CNR with 50 units suggest a buckle size of approximately 4 Å along the centroidal axis. In the Chebyshev–Ritz calculations, as the buckling number increases for a fixed length, the calculated buckle size decreases. For a CNR with a length of 200 units, the estimated buckle size reaches about 4 Å at a buckling number of 56, suggesting that exceeding this point would result in unrealistically small buckles. Such a reduction would lead to excessively altered C-C bond lengths, diverging significantly from realistic configurations. Thus, the Chebyshev–Ritz method results align with the MD simulation shown in Figure 2b, which shows no obvious regular buckled structure. This indicates that at a length of 200 units, the structure is unlikely to adopt a regular buckled mode. The absence of local minima suggests that the nanoring does not experience the same mechanical instabilities as in shorter rings and thus remains in an unbuckled or irregularly deformed state.

### 3.3. Structural Transitions of CNRs Analyzed by Chebyshev–Ritz and MD Methods

**Figure 4:**
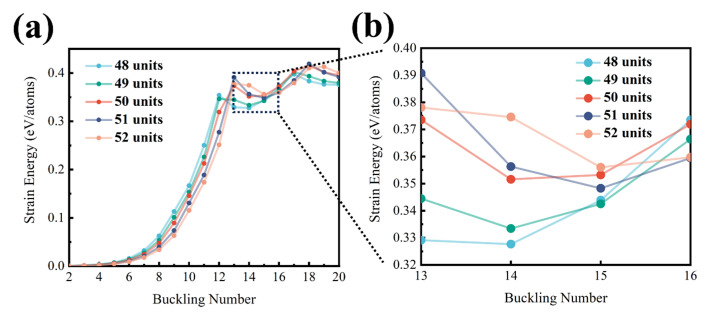
(**a**) The structural transition of CNRs at different lengths. (**b**) A zoomed-in image of the dashed region. The blue, green, red, navy, and orange curves represent the corresponding strain energy per atom calculated by the Chebyshev–Ritz method with lengths of 48, 49, 50, 51, and 52 units, respectively.

**Figure 5:**
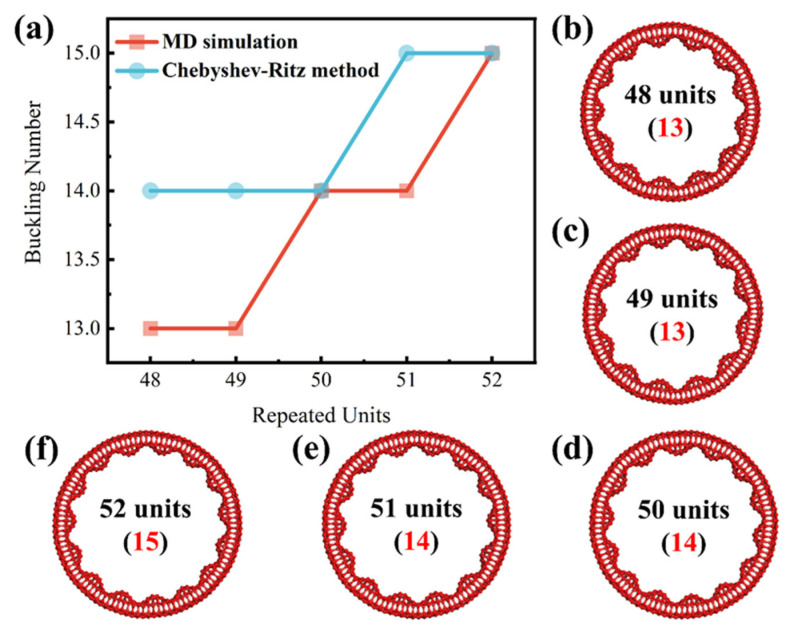
(**a**) A comparison between the Chebyshev–Ritz method and MD simulation. The red and bule lines represent the buckling number of CNRs at different lengths resulted from the MD and Chebyshev methods, respectively. (**b**–**f**) The equilibrated morphologies obtained using the MD simulations with different lengths of 48, 49, 50, 51, and 52 units, respectively. The corresponding buckling numbers are marked in red.

While the mechanical parameters used for CNRs with 50 units have been fixed, the study of structural transitions with increasing CNR units remains underexplored. The sequence of symmetrical transitions from *C_nh_* to either *C*_(*n*−1)*h*_ or *C*_(*n*+1)*h*_ can be investigated using the Chebyshev–Ritz method. As shown in Figure 4, the strain energy per atom is calculated from five different sequential unit lengths, which are 48, 49, 50, 51, and 52 units, respectively. The equilibrium structure corresponds to the local minimum of the curve, indicating that a structural transition occurs at 50 units, shifting from a buckling number of 14 to 15. MD simulations also show such structural transitions with different buckling numbers. As illustrated in Figure 5a, the CNR equilibrium structure undergoes a transition at a length of 49 units, shifting from a buckling number of 13 to 14. As the length increases, another transition occurs at 51 units, changing from a buckling number of 14 to 15. The equilibrated morphologies obtained using the MD simulations with the discussed lengths are illustrated in Figure 5b–f, respectively. Compared to the Chebyshev–Ritz method, these transitions in the MD simulations occur one unit of length later. Hence, these results demonstrate that both our MD simulations and Chebyshev–Ritz modeling can effectively and accurately capture the regular buckled structures of CNRs with comparable results.

## 4. Conclusions

We conducted a comprehensive comparison between MD simulations and the Chebyshev–Ritz method to investigate the structural properties of CNRs, focusing on the emergence of a regular buckling shape, which occurs at specific toric lengths. Our results reveal that the Chebyshev–Ritz method, when its mechanical parameters are appropriately adjusted, accurately replicates the behavior of systems observed in MD simulations, which are typically used to simulate these CNRs. Both MD simulations and the Chebyshev–Ritz method provide stable and consistent descriptions of CNRs exhibiting regular buckled structures and effectively capture transitions in structural configurations across different buckling numbers. The Chebyshev–Ritz method offers a versatile toolkit for analyzing nanomaterials with diverse geometric configurations, accommodating systems of any size without the computational limitations inherent to MD simulations. It compares favorably with MD simulations in terms of reliability and precision, especially when experimental mechanical constants are carefully matched. This flexibility allows for the accurate deduction of mechanical properties that match experimental observations, underscoring the method’s reliability and precision. These findings highlight the Chebyshev–Ritz method as a powerful alternative for studying complex nanostructures, with potential applications across a wide range of nanoscale systems.

## Data Availability

Data will be made available on request.
